# A serological survey of SARS-CoV-2 in cat in Wuhan

**DOI:** 10.1080/22221751.2020.1817796

**Published:** 2020-09-17

**Authors:** Qiang Zhang, Huajun Zhang, Jindong Gao, Kun Huang, Yong Yang, Xianfeng Hui, Xinglin He, Chengfei Li, Wenxiao Gong, Yufei Zhang, Ya Zhao, Cheng Peng, Xiaoxiao Gao, Huanchun Chen, Zhong Zou, Zheng-Li Shi, Meilin Jin

**Affiliations:** a National Key Laboratory of Agricultural Microbiology, Huazhong Agricultural University, Wuhan, People’s Republic of China; b College of Veterinary Medicine, Huazhong Agricultural University, Wuhan, People’s Republic of China; c Key Laboratory of Development of Veterinary Diagnostic Products, Ministry of Agriculture, Wuhan, People’s Republic of China; d CAS Key Laboratory of Special Pathogens, Wuhan Institute of Virology, Center for Biosafety Mega-Science, Chinese Academy of Sciences, Wuhan, People’s Republic of China

**Keywords:** COVID-19, SARS-CoV-2, serological investigation, cats, serum antibody dynamic

## Abstract

COVID-19 is a new respiratory illness caused by SARS-CoV-2, and has constituted a global public health emergency. Cat is susceptible to SARS-CoV-2. However, the prevalence of SARS-CoV-2 in cats remains largely unknown. Here, we investigated the infection of SARS-CoV-2 in cats during COVID-19 outbreak in Wuhan by serological detection methods. A cohort of serum samples were collected from cats in Wuhan, including 102 sampled after COVID-19 outbreak, and 39 prior to the outbreak. Fifteen sera collected after the outbreak were positive for the receptor binding domain (RBD) of SARS-CoV-2 by indirect enzyme linked immunosorbent assay (ELISA). Among them, 11 had SARS-CoV-2 neutralizing antibodies with a titer ranging from 1/20 to 1/1080. No serological cross-reactivity was detected between SARS-CoV-2 and type I or II feline infectious peritonitis virus (FIPV). In addition, we continuously monitored serum antibody dynamics of two positive cats every 10 days over 130 days. Their serum antibodies reached the peak at 10 days after first sampling, and declined to the limit of detection within 110 days. Our data demonstrated that SARS-CoV-2 has infected cats in Wuhan during the outbreak and described serum antibody dynamics in cats, providing an important reference for clinical treatment and prevention of COVID-19.

## GRAPHICAL ABSTRACT

**Figure:**
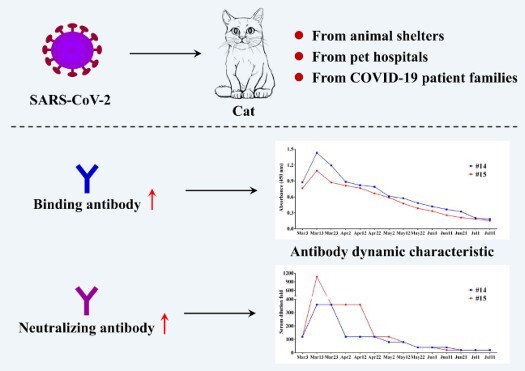

## Introduction

In December, 2019, an outbreak of pneumonia of unknown cause occurred in Wuhan, China. The pathogen was soon identified to be the severe acute respiratory syndrome coronavirus 2 (SARS-CoV-2), and the disease was designated coronavirus disease 2019 (COVID-19) by World Health Organization (WHO) [1,2]. The clinical symptoms of COVID-19 mainly include asymptomatic infection, mild-to-severe respiratory tract illness, and even death [3]. Compared with SARS-CoV, SARS-CoV-2 has the higher basic reproduction number, representing more transmissibility [4]. Within a very short period of time, COVID-19 has quickly become a very serious threat to travel, commerce, and human health worldwide[5]. By 24 July 2020, a total of 15,012,731 confirmed cases, including 619,150 deaths, involving 216 countries, areas, or territories, have been reported globally by WHO (https://www.who.int/emergencies/diseases/novel-coronavirus-2019).

The outbreak of COVID-19 was first confirmed in Wuhan, China, possibly associated with a seafood market. However, so far, there is no evidence that the seafood market is the original source of SARS-CoV-2 [6]. Before SARS-CoV-2, four types of beta coronaviruses can infect humans, including SARS-CoV and MERS-CoV which are highly pathogenic and both originated from bats [7,8]. Genome analysis showed that SARS-CoV-2 has 96.2% overall genome sequence identity with Bat CoV RaTG13, indicating that SARS-CoV-2 could also originate from bats [9]. The transmission of SARS-CoV-2 from bats to humans was suspected to via the direct contact between humans and intermediate host animals [6]. Although several coronaviruses related to SARS- CoV-2 were isolated from pangolin, the molecular and phylogenetic analyses showed that SARS-CoV-2 hardly emerged directly from this pangolin-CoV-2020 [10]. At present, it remains largely unknown which animals were the intermediate host of SARS-CoV-2. Our previous study showed that SARS-CoV-2 uses the same cell entry receptor, angiotensin converting enzyme II (ACE2), as SARS-CoV [9], suggesting that SARS-CoV-2 has the same host range as SARS-CoV. Previous report demonstrated that SARS-CoV can infect ferrets and cats [11], implying that they might be also susceptible to SARS-CoV-2. In fact, the recent reports have shown that SARS-CoV-2 can indeed infect cats, but not cause any obvious symptoms [12–14].

Cat is one of the most popular pets and often has close contact with humans. Thus, the prevalence of SARS-CoV-2 in cats is very important to investigate, especially in outbreak regions. Here, we investigated the serological prevalence of SARS-CoV-2 in cats by an indirect ELISA and virus neutralization tests (VNT), and monitored the serum antibody dynamics of cats infected SARS-CoV-2, providing a basis for further understanding the infection of SARS-CoV-2 in cats.

## Methods

### Sample collection

A total of 102 cats were sampled in Wuhan between Jan. and Mar. 2020 with three sources: (1) 46 abandoned cats were from 3 animal shelters, (2) 41 cats were from 5 pet hospitals, and (3) 15 cats were from COVID-19 patient families. All cats in shelter and hospital were live in relatively close cages. Blood samples were collected via leg venipuncture and sera were separated and stored at −20°C until further processing. Nasopharyngeal and anal swabs were collected and put into tubes containing viral transport medium-VTM (Copan Diagnostics, Brescia, Italy) [15]. All samples were collected under full personal-protective equipment, including head covers, goggles, N95 masks, gloves, and disposable gowns. A set of 39 cat sera were retrieved from the serum bank in our lab, which were collected from Wuhan between Mar. and May, 2019. Hyperimmune sera were obtained from Neuropathy Pathogen Laboratory, Huazhong Agriculture University, with neutralization titres of 1/640 and 1/1280, respectively, against type I and II feline infectious peritonitis virus (FIPV). The convalescent serum of a COVID-19 patient was collected from Jiangxia Tongji hospital with the consent of the patient and a neutralization titre 1/1280.

### Virus and cells

SARS-CoV-2 (IVCAS 6.7512) was isolated from a COVID-19 patient as previously described[9]. Vero E6 was purchased from ATCC (ATCC^®^ CRL-1586^™^).

### Enzyme-linked immunosorbent assay (ELISA)

Antibody was tested by indirect ELISA with the SARS-CoV-2 RBD protein (Sino Biological Inc., China) and peroxidase conjugated goat anti-cat IgG (Sigma-Aldrich, USA). Brieﬂy, ELISA plates were coated overnight at 4°C with RBD protein (1 μg/ml, 100 μl per well). After blocked with PBS containing 5% skim milk for 2 h at 37°C, the plates were added with sera at a dilution of 1: 40. After incubation for 30 min at 37°C, the plates were washed five times with washing buffer (PBS containing 0.05% Tween-20). A 1:20,000 diluted anti-cat IgG was added and incubated for an additional 30 min. After another 5 washes, TMB Substrate (Sigma-Aldrich, USA) was added and incubated for 10 min. Then the reaction was stopped, and optical density (OD) was measured at 450 nm. As the judgment method described previously[16, 17], those sera were considered positive if the OD values were twice higher than the mean OD of the 39 sera collected between Mar. and May, 2019.

### VNT

For virus neutralization test, serum samples were heat-inactivated by incubation at 56°C for 30 min. Each serum sample was serially diluted with Dulbecco’s Modified Eagle Medium (DMEM) as two fold or three fold according to the OD value and the sample quality, mixed with equal volume of diluted virus and incubated at 37°C for 1 h. Vero E6 cells in 24-well plates were inoculated with the sera-virus mixture at 37°C; 1 h later, the mixture was replaced with DMEM containing 2.5% FBS and 0.8% carboxymethylcellulose. The plates were fixed with 8% paraformaldehyde and stained with 0.5% crystal violet 3 days later. All samples were tested in duplicate and neutralization titres were defined as the serum dilution resulting in a plaque reduction of at least 50% [18].

### Western blotting assay

The total protein concentration of purified and inactivated SARS-CoV-2 was determined by Bradford protein assay [19]. 4 μg protein was subjected to 8% sodium dodecyl sulfate-polyacrylamide gel electrophoresis (SDS-PAGE) and transferred on to nitrocellulose membrane. Then viral proteins were blotted with cat sera or human convalescent serum. Protein bands were visualized by incubation with a goat anti-cat IgG or mouse anti-human IgG and then detected using the ECL System (Amersham Life Science, Arlington Heights, IL, USA).

## Results

### The detection of binding antibody against SARS-CoV-2 in cats

**Figure 1.**
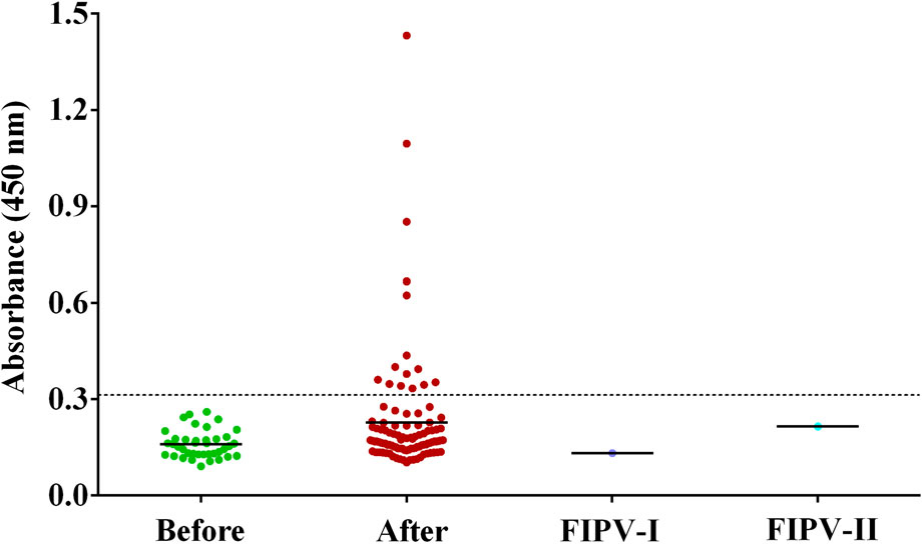
ELISA of cat serum samples against the recombinant receptor binding domain (RBD) of SARS-CoV-2 spike protein. The dashed line is the cut-off. Each dot represents one individual sample within each antigen panel. Before, serum samples collected between Mar. and May, 2019 prior to COVID-19 outbreak; After, serum samples collected between Jan to Mar., 2020 after COVID-19 outbreak; FIPV- I, hyperimmune sera against type I feline infectious peritonitis virus (FIPV); FIPV-II, hyperimmune sera against type II FIPV.

Cat serum samples were detected with an indirect ELISA based on recombinant RBD protein. From the 39 prior-to-outbreak sera, whose optical density (OD) varied from 0.091 to –0.261, we set the cut-off as 0.32. The positive samples of 102 cat sera were screened according to this standard. As shown in Table 1 and Figure 1, 15 (14.7%) cat sera collected during the outbreak were positive, with five strong positive ones with OD more than 0.6. Of which, Cat#14 and Cat#15 were from the same owner who was COVID-19 patient. Both type I and II FIPV hyperimmune sera showed no cross-reactivity with SARS-CoV-2 RBD protein.

**Table 1.** Detection of the antibodies of SARS-CoV2 in cats.

Cat NO.	ELISA (OD450)	Neutralization titre	Background of cats
#1	0.353	1/40	from pet hospital
#2	0.334	1/80	abandoned cat
#3	0.348	1/40	abandoned cat
#4	0.687	1/360	COVID-19 patient owner
#5	0.394	1/40	from pet hospital
#6	0.401	–	from pet hospital
#7	0.379	–	from pet hospital
#8	0.345	1/20	from pet hospital
#9	0.351	1/40	from pet hospital
#10	0.624	1/20	abandoned cat
#11	0.342	1/40	abandoned cat
#12	0.852	–	abandoned cat
#13	0.437	–	abandoned cat
#14	1.432	1/360	COVID-19 patient owner
#15	1.095	1/1080	COVID-19 patient owner

– indicates under the limit of detection.

### High levels of neutralizing antibody against SARS-CoV-2 were found in infected cats

**Figure 2.**
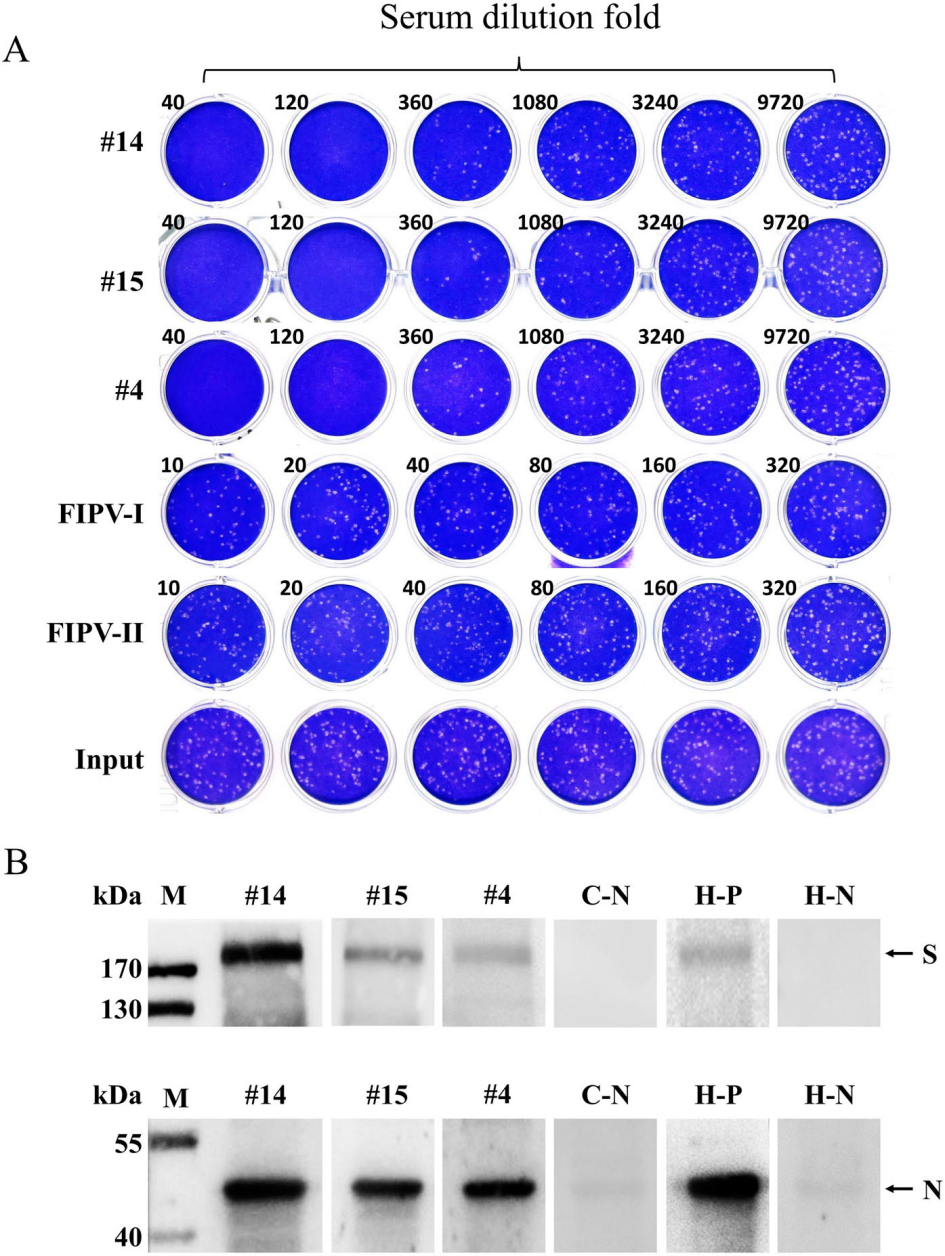
Virus neutralization test and Western blot assay of cat serum samples for SARS-CoV-2 (A) Cat#14, Cat#15 and Cat#4 sera were 3-fold serially diluted and mixed with SARS-CoV-2; after incubated at 37°C for 1 h, the mixture was used to infect Vero E6 cells, and replaced with semi-solid media 1 h later. The plates were fixed and stained 3 days later. All samples were tested in duplicate. (B) Western blot of purified SARS-CoV-2 with cat or human sera. All sera were diluted 100 folds. C-N, negative cat serum. H-P, human convalescent serum. H-N, healthy human serum.

To further conﬁrm the presence of SARS-CoV-2 speciﬁc antibody in cats, all of 15 ELISA-positive sera were subjected to VNTs for SARS-CoV-2. Among them, 11 (10.8%) had SARS-CoV-2 neutralizing antibodies with a titre ranging from 1/20 to 1/1080 (Table 1 and Figure 2(A)). However, 4 sera including #12, which was ELISA strong positive with OD of 0.852, showed no neutralizing activity, most likely because of recognition of non-neutralizing epitopes. Another ELISA strong positive one, #10, had very weak neutralizing activity. But strong neutralization was observed for the other three ELISA strong positive sera, namely #4, #14 and #15, with neutralizing titre of 1/360–1/1080. Consistent with the high neutralizing titre, the owners of Cat#4, Cat#14 and Cat#15 were diagnosed as COVID-19 patients. Cat#1, Cat#5∼9 was from pet hospitals, while Cat#2, Cat#10∼13 were initially abandoned cats and kept in animal protection shelters after the outbreak. Again, both type I and II FIPV hyperimmune sera were negative for VNT.

### The sera of infected cats can specifically bind the S and N proteins of SARS-CoV-2

Western blot assay was also performed to further verify the existence of SARS-CoV-2 speciﬁc IgG in cat serum. As shown in Figure 2(B), S and N proteins of the purified SARS-CoV-2 were successfully detected with #4, #14 and #15 sera after diluted 100 folds, as well as human convalescent serum[20]. Conversely, the ELISA negative cat serum and healthy human serum did not probe the protein bands, thereby demonstrating the existence of SARS-CoV-2 speciﬁc IgG in cat serum.

### The dynamic characteristic of serum antibody against SARS-CoV-2 in cats

**Figure 3.**
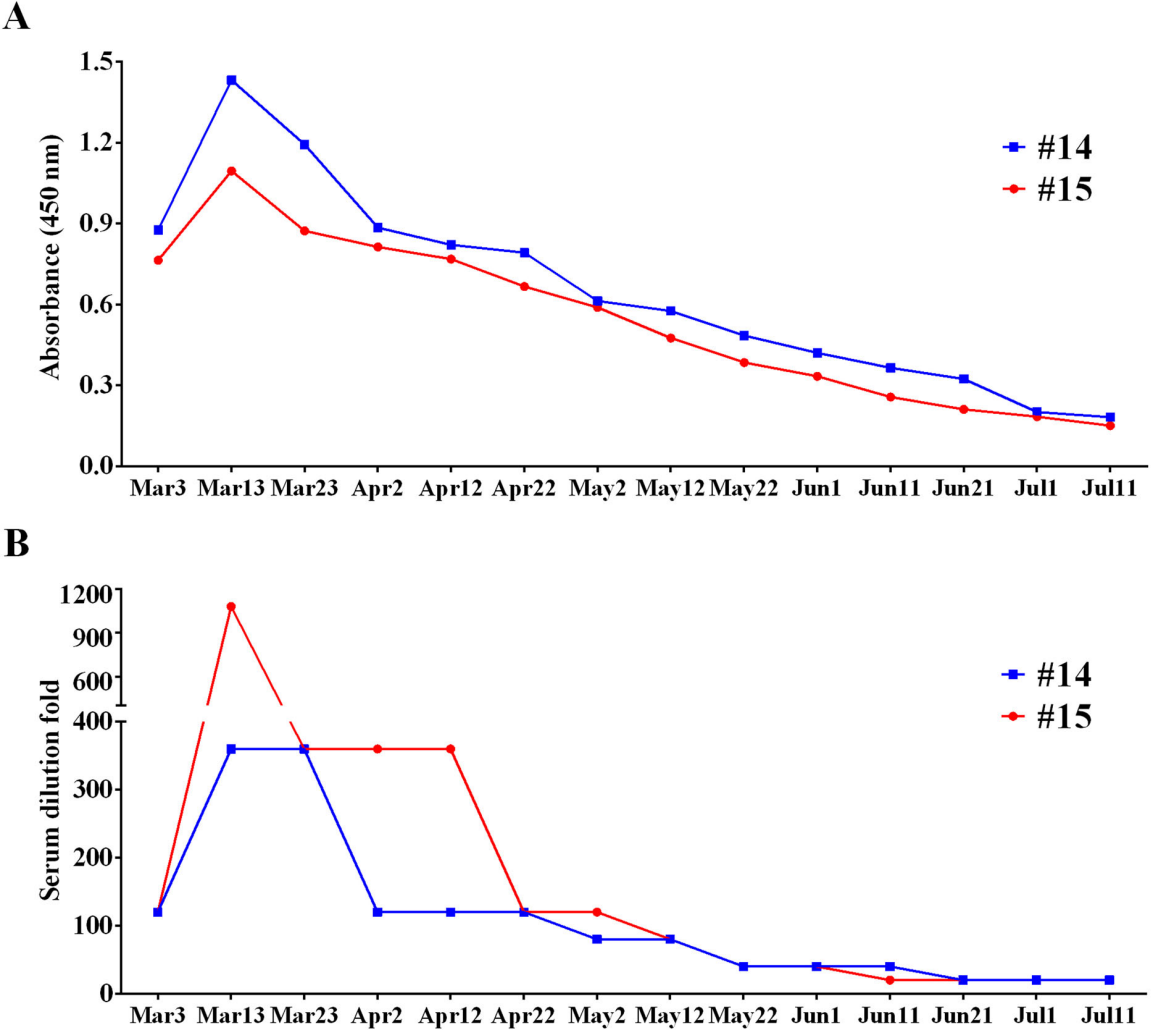
The dynamic change of cat serum antibody for SARS-CoV-2. (A) ELISA detection and (B) neutralization test of cat serums. The serums of Cat#14 and Cat#15 were collected every 10 days from Mar. 3 to Jul. 11. Then the ELISA against SARS-CoV-2 RBD and the virus neutralization test were performed.

Fortunately, we had access to two cats, Cat#14 and Cat#15, for a long time, which gave us the opportunity to track the dynamic of antibody. We continuously sampled Cat#14 and Cat#15 every 10 days over 130 days. As shown in Figure 3(A), RBD antibodies of these two cats reached the peak at the second sampling, when both showed OD>1.0 for ELISA. After that, RBD antibodies turned down and decreased to detection limit in 110 days. Accordingly, neutralizing antibodies showed similar trend (Figure 3(B)).

## Discussion

In this study, we detected the presence of SARS-CoV-2 antibodies in cats in Wuhan during the COVID-19 outbreak with ELISA, VNT and western blot. A total of 102 cats were tested, 15 (14.7%) were positive for RBD based ELISA and 11 (10.8%) were further positive with VNT. These results demonstrated that SARS-CoV-2 has infected cats in Wuhan, implying that this risk could also occur at other outbreak regions. In fact, it has been indeed successively reported that SARS-CoV-2 infected cats under natural conditions [14,21]. Retrospective investigation confirmed that all of ELISA positive sera were sampled after the outbreak, suggesting that the infection of cats could be due to the virus transmission from humans to cats. Certainly, it is still needed to be verified via investigating the SARS-CoV-2 infections before this outbreak in a wide range of sampling. At present, there is no evidence of SARS-CoV-2 transmission from cats to humans. However, a latest report shows that SARS-CoV-2 can transmit between cats via respiratory droplets [12]. Over all, some preventive measures are necessary for blocking the human-to-cat transmission or preventing the potential transmission risk of cats to other animals or humans.

Through analysing the background of the tested cats, we found that 4 of abandoned cats (9.8%), 4 of cats from pet hospitals (8.7%), and 3 of cats with patient owners (20%) were positive with VNT. Although the positive rate among different source cats had no significant differences, the three cats with the highest neutralization titres (1/1080, 1/360, and 1/360, respectively) were owned by COVID-19 patients. On the contrary, the sera collected from pet hospital cats and stray cats had neutralizing activity of 1/20–1/80, indicating that the high neutralization titres could be due to the close contact between cats and COVID-19 patients. In addition, our data demonstrated that the duration of neutralizing antibody against SARS-CoV-2 is relatively transient in the infected cats. So, the low neutralization titres could also be due to the long-time interval between sample collection and actual infection date. Although the infection in stray cats was not fully understood, it is reasonable to speculate that these infections are probably due to the contact with SARS-CoV-2 polluted environment, or COVID-19 patients who fed the cats.

The antibody-mediated humoral response is crucial for preventing viral infections, of which the neutralizing antibody can reduce the entry of the virus into an infected cell via blocking the interaction between virus and cell [22]. So, the neutralizing antibody is an important indicator that can reflect the host antiviral ability. Although numerous reports have indicated that the infection of SARS-CoV-2 can induce the production of neutralizing antibody, the understanding about the dynamics of SARS-CoV-2 neutralizing antibody remains largely unknown. Here, we continuously monitored the dynamics of binding antibody and neutralizing antibody against SARS-CoV-2 in the infected cats. We found that both these two antibodies can be induced with a relatively high level, however the duration of peak titre was very short, and decreased to the limit of detection within 110 days. It was worth noting that, these two cats were from the same owner who presented with fever and cough in mid-February, and was diagnosed and segregated as COVID-19 patient on February 21. Then these two cats were fostered in pet hospital and were also segregated. Combined with the dynamic characteristic and timeline of antibody response, we speculated that these two cats should be infected at the same time. In addition, considering that the two cats were constantly in segregation, we believed that our data represented the antibody dynamic characteristic of primary infection. Importantly, this transient antibody response induced by SARS-CoV-2 resembles those observed in seasonal coronavirus infections, implying that the convalescent cats after SARS-CoV-2 infection remain the risk of re-infection. In fact, this similar transient antibody response has also been observed in human antibody [23,24], suggesting that cat has a great potential as an animal model for assessing the characteristic of antibody against SARS-CoV-2 in human. Our data provided a very important reference for the clinical treatment and prevention of COVID-19.

In addition, we also collected nasopharyngeal and anal swabs of each cat, and conducted SARS-CoV-2 specific qRT-PCR using a commercial kit which targeted *ORF1ab* and *N* genes. Seven samples from five cats were N gene single positive with CT ranging from 34.9 to 36.7, but no double gene positive sample was detected (according to the manufacture instruction in which CT value less than 37 was deemed as positive). The reason might be (1) that the viral RNA load is too low to be detected; (2) the period that cat shed SARS-CoV-2 may be very short[21], along with asymptomatic infection, we didn’t catch the moment of acute infection; (3) there may be variants in the genomic sequences in cats, leading to the failure in amplification in cat samples.

In conclusion, our study provided serological evidence for SARS-CoV-2 infection in pets, and described the dynamic characteristic of serum antibody in cats. Further research is needed to investigate the transmission route of SARS-CoV-2 from humans to cats. In addition, some preventive measures should be implemented to maintain a suitable distance between COVID-19 patients and companion animals such as cats and dogs, and hygiene and quarantine measures should also be established for those high-risk animals.
